# Subacute pain trajectories following major musculoskeletal surgery in adolescents: A pilot study

**DOI:** 10.1080/24740527.2020.1765692

**Published:** 2020-12-30

**Authors:** Jennifer A. Rabbitts, Cornelius B. Groenewald, Chuan Zhou

**Affiliations:** a Department of Anesthesiology & Pain Medicine, University of Washington, Seattle, Washington, USA; b Center for Clinical and Translational Research, Seattle Children’s Hospital, Seattle, Washington, USA; c Center for Child Health, Behavior and Development, Seattle Children’s Hospital, Seattle, Washington, USA; d Department of Pediatrics, University of Washington, Seattle, Washington, USA

**Keywords:** subacute pain, pain trajectory, chronic postsurgical pain, adolescent, musculoskeletal surgery, musculoskeletal pain, acute to chronic pain

## Abstract

**Background**: Adolescents who undergo major surgery experience high rates of disabling acute and chronic postsurgical pain (CPSP). However, little is known about the subacute period when acute to chronic pain transition occurs.

**Aims**: The aims of this study were to examine feasibility of electronic daily monitoring of pain and opioid use in adolescents during the first 30 days at home following major surgery and identify target features of subacute pain curves associated with CPSP at 4 months.

**Methods**: Twenty-five youth (10–18 years) undergoing major musculoskeletal surgery reported daily pain severity, interference, and opioid use on the Brief Pain Inventory each evening for 30 days after hospital discharge to form short time series trajectories. At 4 months, youth reported on pain intensity and health-related quality of life. Characteristics of subacute pain severity and interference curves were compared by 4-month CPSP status.

**Results**: At 4 months, 20.8% of youth met criteria for CPSP. During the 30-day monitoring period, youth who went on to develop CPSP reported high pain severity on 45.9% of days compared to 2.9% of days in youth who recovered (*P* = 0.005) and high pain interference on 49.4% of days vs. 9.7% in youth who recovered (*P* = 0.01). Pain variability and rate of change were not significantly associated with CPSP in our pilot sample.

**Conclusions**: We found it feasible to collect daily pain data in youth recovering at home after major surgery. Pilot findings suggest that daily electronic monitoring may identify early recovery problems at home after surgery. Larger studies are needed to validate subacute pain trajectory features to identify risk for CPSP.

## RÉSUMÉ

**Contexte**: Les adolescents qui subissent des interventions chirurgicales majeures présentent des taux élevés de douleur chronique postopératoire aiguë et débilitante. Cependant, on sait peu de choses sur la période subaiguë, pendant laquelle la transition de la douleur aiguë à la douleur chronique s’effectue.**Objectifs**: Cette étude avait pour but d’examiner la faisabilité d’un suivi électronique quotidien de la douleur et de l’utilisation d’opioïdes chez les adolescents au cours des 30 premiers jours à la maison après une opération chirurgicale importante, ainsi que de déterminer les caractéristiques cibles des courbes de douleur subaiguë associées à la douleur postopératoire chronique après quatre mois.**Méthodes**: Vingt-cinq jeunes (10-18 ans) subissant une chirurgie musculo-squelettique majeure ont déclaré chaque jour l’intensité et l’interférence de leur douleur, ainsi que l’utilisation d’opioïdes à l’aide de l’Inventaire abrégé de la douleur chaque soir pendant 30 jours après leur sortie de l’hôpital, afin de former de courtes trajectoires en série chronologique. Au bout de quatre mois, les jeunes ont déclaré l’intensité de leur douleur et de leur qualité de vie liée à la santé. Les caractéristiques des courbes portant sur l’intensité de la douleur subaiguë et l’interférence ont été comparées en fonction du statut relatif à la douleur postopératoire chronique au bout de quatre mois.**Résultats**: Au bout de quatre mois, 20,8 % des jeunes répondaient aux critères de la douleur chronique postopératoire. Au cours de la période de suivi de 30 jours, les jeunes qui ont ensuite développé de la douleur chronique post-opératoire ont fait état d’une douleur très intense dans 45,9 % des jours, comparativement à 2,9 % des jours chez les jeunes qui se sont rétablis (P = 0,005). Ils ont aussi déclaré une interférence de la douleur élevée dans 49,4 % des jours comparativement à 9,7 % chez les jeunes qui se sont rétablis (P = 0,01). La variabilité de la douleur et le taux de changement n’étaient pas significativement associés à la douleur chronique postopératoire dans notre échantillon pilote.**Conclusions**: Nous avons constaté qu’il était possible de recueillir des données quotidiennes sur la douleur chez les jeunes qui se rétablissent à la maison après une chirurgie. Les résultats du projet pilote indiquent que la surveillance électronique quotidienne pourrait permettre de repérer les problèmes de rétablissement précoces à la maison après l’opération. Des études de plus grande envergure sont nécessaires pour valider les caractéristiques de la trajectoire de la douleur subaiguë afin de déterminer les risques de douleur chronique postopératoire.

## Introduction

Adolescents who undergo major musculoskeletal surgery experience high rates of intense acute postoperative pain^1^ and ~20% go on to transition to chronic postsurgical pain (CPSP).^2–4^ Postoperative pain is a source of significant distress for these youth,^5^ with impact on both short-term^6^ and long-term postsurgical outcomes.^7^ Though knowledge is increasing regarding acute and chronic postsurgical pain in this population, little is known about the *subacute* period when, by definition, acute to chronic pain transition occurs. Understanding of early risk for transition to CPSP is critical for early intervention efforts.^8^

Prior studies have described long-term trajectories of postsurgical pain in youth, identifying distinct patterns of pain over the first year after surgery and highlighting trajectories that lead to chronic pain and disability.^4,7,9^ However, recent studies suggest that more granular methods for studying the early transition from acute pain to CPSP may better inform intervention. For example, a study in adults examined feasibility of daily monitoring for 30 and 60 days following knee/hip arthroplasty and cesarean delivery, respectively, demonstrating feasibility of daily assessment through high completion rates among adults following major surgery.^10^ This study found high variability between individuals’ pain resolution and identified a change point in the pattern of pain during the first 10 to 21 days following surgery. In adolescents, two studies have identified postoperative pain 2 weeks following major surgery as predictive of chronic pain outcomes,^3,7^ including our own study examining long-term trajectories, which found that youth who followed an unfavorable trajectory were already identifiable at 2 weeks.^7^ This suggests that the initial 2 weeks following surgery may be a critical time period when processes that contribute to pain resolution vs. transition to chronic pain may develop. However, prior studies have not examined pain trajectories during these initial days to weeks of recovery, and the feasibility of daily monitoring immediately following discharge home from major surgery is unknown. These gaps in knowledge have hindered early detection of recovery problems necessary to implement early intervention before adverse trajectories are established.

In addition to CPSP, researchers have identified persistent postsurgical opioid use as a significant concern among adolescents.^11^ Opioids remain the cornerstone of acute postoperative pain management following major surgery; however, the potential negative consequences of legitimate opioid use in the context of the opioid epidemic are increasingly being recognized. Indeed, in addition to persistent opioid use, postsurgical opioid use may lead to opioid misuse later in life.^12^ Furthermore, opioids are often overprescribed following surgery, resulting in a large reservoir of leftover opioids at home.^13^ Leftover opioids are a common source for adolescent opioid diversion and misuse.^14^ In response to these concerns, health care agencies have advocated for reduced opioid prescribing to adolescents.^15^ However, patterns of opioid use at home after major surgery and their relation to problematic opioid use behaviors in adolescents are unknown.

The aims of this pilot study were to examine feasibility of electronic daily monitoring to characterize subacute pain and opioid use in adolescents during the first 30 days at home following major musculoskeletal surgery. Further we aimed to identify target features of raw subacute pain curve data associated with CPSP at 4 months.

## Materials and Methods

### Setting and Population

This study was conducted at a pediatric specialty hospital situated in the northwestern United States. Youth enrolled in an ongoing longitudinal study examining psychosocial predictors of acute and chronic postsurgical pain were eligible for participation in this pilot study.^29^ Inclusion criteria for the larger study were (1) age 10 to 18 years of age, (2) able to read and understand English, and (3) scheduled to undergo a major musculoskeletal surgery (spinal fusion, Nuss or Ravitch procedure, hip or femur osteotomy) for an idiopathic deformity. Prior research has shown similar rates of CPSP across these major musculoskeletal surgery types.^7^ Exclusion criteria included (1) a comorbid medical condition requiring a daily medication (except preventive medication for asthma on seasonal allergies), (2) mental health condition receiving active treatment, (3) diagnosed chronic pain condition, (4) prior major surgery (open surgery or major musculoskeletal surgery), or (5) developmental delay. There were no additional exclusion criteria for participation in the pilot study.

### Procedures

Institutional review board approval was obtained for all study procedures (IRB ID: STUDY00000039). Potentially eligible participants for the larger study were identified from surgery clinic and procedure schedules and were sent a mailing containing a study flyer and consent documents. Research assistants subsequently contacted potential participants by phone, or in presurgery clinic if unable to reach by phone, to explain the studies and screen interested patients. During the 23-month enrollment time frame of this pilot study, youth who enrolled in the larger study were provided information about the additional pilot study.

Forty youth were approached for potential participation, of whom 25 enrolled (participation rate 63%). The 15 families who declined all cited the time commitment of the larger study as the reason for nonparticipation in this additional study. Parents and youth (18 years of age) from interested families provided their written informed consent to participate and youth <18 years provided informed assent to participate.

The larger study procedures include four assessments over the year following surgery: presurgery, and 2 weeks, 4 months, and 12 months postsurgery. Participants who enrolled into the pilot study completed a 30-day daily assessment following hospital discharge; there were no additional procedures for these participants.

During the week before surgery, parents completed a demographic questionnaire and reported their child’s current medications, and youth reported on presurgery pain intensity and health-related quality of life (HRQOL). Research assistants monitored participants’ medical records daily to identify discharge dates. Starting the day following hospital discharge, youth completed a daily self-report pain measure for 30 days. At 4 months postsurgery, youth completed self-report pain and health outcomes measures; parents reported their child’s current medications. All data were collected using REDCap electronic data tools hosted at the Institute of Translational Health Sciences.^17^ Survey links for daily monitoring and outcomes measures were sent to participants’ e-mails via REDCap, with automated REDCap e-mail reminders sent 3 h after daily measures and 3 days after survey measures, if these were not yet completed. Diaries were also checked on days 1 and 3 and weekly thereafter, with additional contact made by mode of family preference (text, phone, e-mail) to encourage completion. Youth received up to $1 incentive per completed daily measure at the end of the 30 days of daily monitoring in the form of gift cards to local or online stores. The retention rate through follow-up was 96%, with one participant dropping out of the study before the 4-month follow-up.

### Measures

#### Sociodemographics

Parents reported on child sex, age, race, and ethnicity, as well as parent education level and family income.

### 30 Days of Daily Monitoring

#### Daily Pain Severity and Pain Interference

Youth completed the short version of the Brief Pain Inventory assessing daily pain severity and pain interference in the preceding 24 h. The Pain Severity Scale comprises four items assessing worst, least, current, and average pain intensity with responses indicated on an 11-point numeric rating scale with anchors 0 = *no pain* and 10 = *pain as bad as you can imagine*. The Pain Interference Scale contains 11 items assessing pain interference with general activity, mood, walking ability, work (inside and outside the home), relations with other people, sleep, and enjoyment of life. Responses to each item are indicated on an 11-point numeric rating scale with anchors 0 = *does not interfere* and 10 = *completely interferes*. Pain severity and pain interference scores are calculated as the average of the pain intensity and interference items, respectively. This measure was originally developed and tested in adults,^18,19^ showing sensitivity to change.^20^ The measure has been extensively used in adolescents,^21,22^ demonstrating construct validity to assess pain and function.^23^

#### Daily Opioid Use

The Brief Pain Inventory item “What treatments or medications are you receiving for your pain?” was used to assess daily medication use. Responses were selected from a drop-down list with the following options: opioid, acetaminophen/Tylenol, anti-inflammatory medications, and other medications, with a complete list of examples. The main opioid-related outcome was “time to opioid cessation” which was measured as the last day of reported opioid use.^24^ We did not gather information on the number of daily opioid doses consumed.

### Outcome Measures

#### Pain Intensity

Youth reported pain intensity on a single item 11-point numeric rating scale (NRS), with anchors 0 = *no pain* and 10 = *worst pain possible*. NRSs for pain intensity are widely used to assess chronic pain^25,26^ and perioperative pain^27,28,29,32^ in adolescents.

#### Health-Related Quality of Life

Youth self-reported HRQOL on the 15-item (short) form of the Pediatric Quality of Life Inventory, which assesses problems with physical, school, emotional, and social functioning.^30^ Items on this measure are transformed to a 0 to 100 scale, with higher scores indicating higher quality of life. Items are then averaged to yield a total quality of life score. The cut point of <74.9 was used for clinically significant impairment in quality of life based on one standard deviation below the population mean^31^ based on normative data.^30^

### Statistical Analyses

We applied two-sided *t*-tests and chi-square tests to compare baseline demographic variables and surgery procedure type between those who enrolled and those who declined participation in the study. We defined adequate daily measure completion rates as 75% of the 30 days of daily measures complete.

We defined our 4-month outcome, CPSP, in accordance with the modified International Association of the Study of Pain definition of pain associated with impairment in quality of life.^16^ Thus, CPSP was defined at 4 months as a binary variable with 1 = *presence of significant pain* (NRS ≥ 3) *and impairment in HRQOL* (Pediatric Quality of Life Inventory < 74.9); 0 = *otherwise*. Construct validity has been demonstrated through expected cross-sectional associations of CPSP with higher pain interference on activities and greater emotional upset from pain.^29^

**Figure 1.**
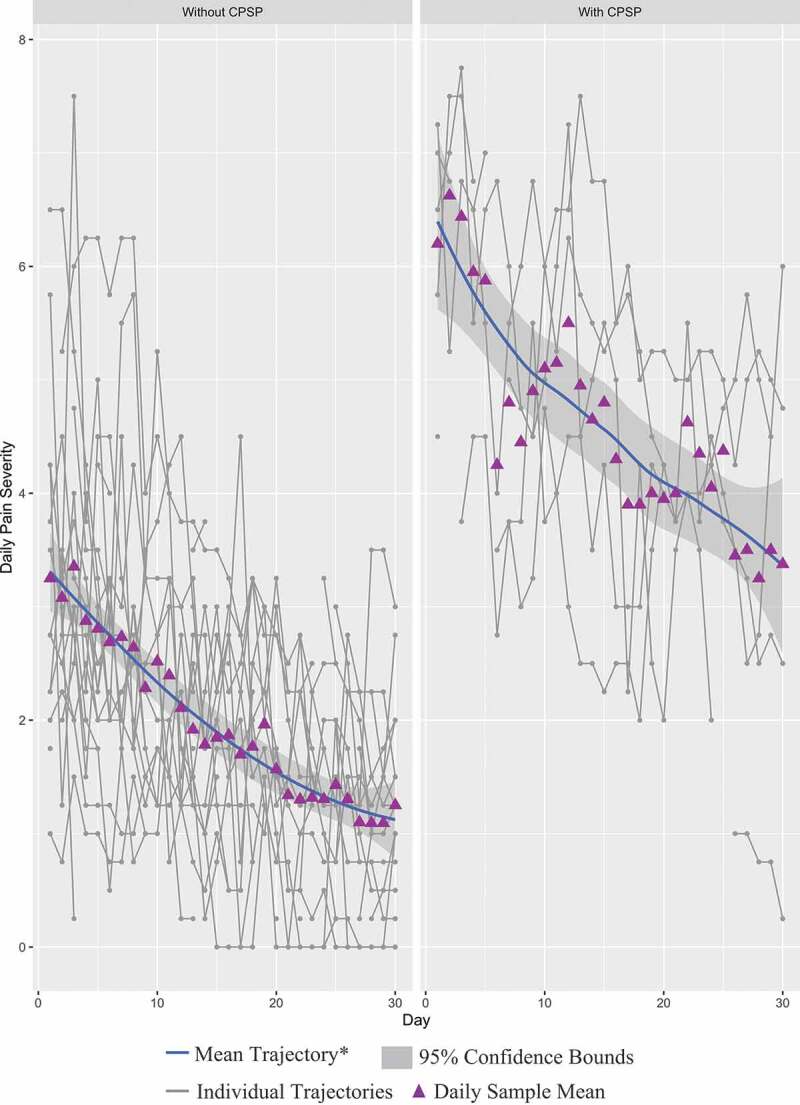
Trajectories of daily subacute pain severity by 4-month chronic postsurgical pain (CPSP) status.^+^*Time to opioid cessation measured as the last day of opioid use.*

**Figure 2.**
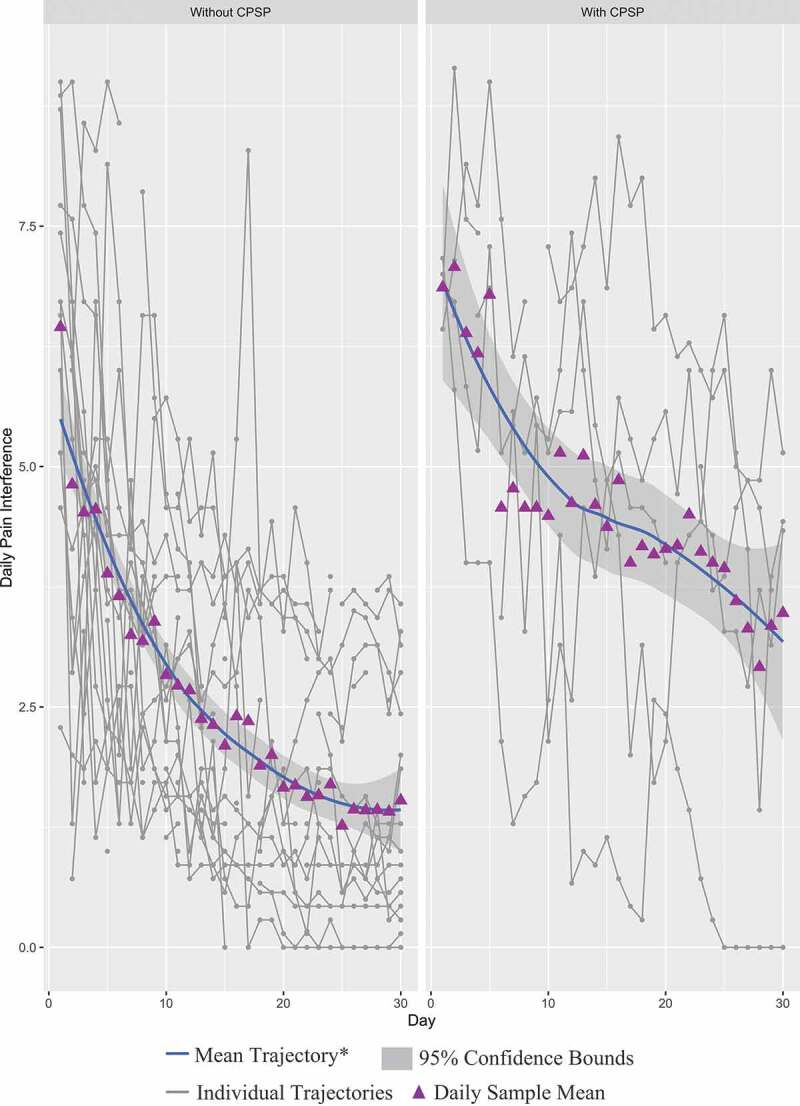
Trajectories of daily subacute pain interference by 4-month chronic postsurgical pain (CPSP) status^*^with locally-weighted scatterplot smoothing (smoothing parameter 0.8)

In total, 631 available person-days of daily data were used in the analyses. Daily assessments of subacute Pain Severity and Pain Interference scales on the Brief Pain Inventory formed short time series trajectories and were examined separately for each participant. For visual examination (Figure 1 and Figure 2), we overlaid individual trajectories with daily averages and a mean trajectory with locally weighted scatterplot smoothing (loess).

We further extracted features, defined a priori, from the raw time series data that capture certain characteristics of the trajectories and that are simple to analyze and interpret. Specifically, features describing magnitude included mean of the first 2 weeks, mean of the entire 30 days, percentage of days with high pain ratings (≥5).^32^ Features describing variability included standard deviation (SD) for the first 2 weeks, SD for the 30-day period, and range (max–min) of data for the 30-day period. Features describing rate of change included slope estimate from fitting a linear regression line to the data and change at the end of 30 days relative to day 1. To account for potential for linear and nonlinear curves, we also extracted difference in slope as a feature, calculated as the difference between the linear slope of the first and the second 15-day periods. A larger difference in slope indicates a more rapid change in early recovery compared to later recovery, whereas a difference of zero indicates a constant slope throughout recovery. Overall, a total of nine features were considered for each process. Descriptive statistics such as means and standard deviations were used to summarize numeric features of the pain severity and pain interference processes across participants.

Time to opioid cessation was examined using survival analysis and presented as a Kaplan-Meier curve for all participants. A log-rank test of equality was used to compare differences in opioid duration between CPSP groups.

Neither presurgery pain intensity, *t*(22) = 0.132, *P* = 0.9, nor HRQOL, *t*(22) = 1.417, *P* = 0.2, was associated with 4-month CPSP status. We therefore applied univariate analyses to compare features of the pain severity process and pain interference process between groups based on CPSP status using two-sample *t*-tests. To account for multiple comparisons, we adjusted *P* values for the between CPSP status comparisons using the Benjamini-Hochberg method to ensure 5% false discovery rate among the rejected hypotheses.^33,34^ All analyses were conducted using R v3.6.1 (R Foundation for Statistical Computing, Vienna, Austria; https://www.R-project.org/).

## Results

### Descriptive Data

Sociodemographic characteristics of the study cohort are presented in Table 1. There were total of 25 participants (21 girls and 4 boys; 80% white). No baseline differences were observed between youth who enrolled compared to youth who declined participation in the study (*P*s > 0.05). No youth were on opioids before surgery. On average youth completed 25 days (84%) of daily assessments during the 30-day monitoring period at home following discharge from surgery. At 4 months, 5 (20.8%) youth met criteria for CPSP, reporting significant pain associated with clinically significant impairment in HRQOL.

**Table 1.** Participant demographic and clinical characteristics

Demographics (*N* = 25)		Mean (range), *n*
Age (years)		14.7 (11.7–18.2)
Sex		
	Female	21 (84%)
	Male	4 (16%)
Child race		
	White	20 (80%)
	Native Hawaiian or other Pacific Islander	1 (4%)
	American Indian or Alaska Native	1 (4%)
	Other/not reported	3 (12%)
Child ethnicity		
	Hispanic or Latino	3 (12%)
	Non-Hispanic or Latino	22 (88%)
Annual household income		
	<$29,999	3 (12%)
	$30,000–$69,999	6 (24%)
	$70,000–$100,000	4 (16%)
	>$100,000	11 (44%)
	Not reported	1 (4%)
Highest parental educational level		
	High school or less	1 (4%)
	Some college/vocational school	4 (16%)
	Bachelor’s degree	13 (52%)
	Graduate/professional	3 (12%)
	Not reported	4 (16%)
Musculoskeletal surgery type		
	Spinal fusion	22 (88%)
	Repair pectus excavatum	3 (12%)

During the 30-day monitoring period, youth reported an average pain score of 2.5 (range 0.8–5.7), with high severity pain (≥5) reported on 12% of days on average (range = 0%–90%). Overall, youth had a relative reduction in pain severity of 52% (interquartile range [IQR] = 37%–68% reduction) over the 30-day period and took an average of 15 days (IQR = 6–21) to report a 50% reduction in pain severity.

In terms of pain interference, youth reported an average pain interference score of 3.0 (range = 1.2–6.7), with interference scores ≥5 reported on 18% of days on average (range = 0%–93%). Overall, youth had a relative reduction in pain interference of 60% (IQR = 38%–71% reduction) over the 30-day period and took an average of 11 days (IQR = 4–16) to report a 50% reduction in pain interference.

Subacute pain severity and interference trajectories along with smoothed mean trajectories are shown in Figures 1 and 2, respectively. Visually, trajectories of youth without CPSP started lower and had more steady improvement over time compared to those of their counterparts with CPSP.

**Figure 3.**
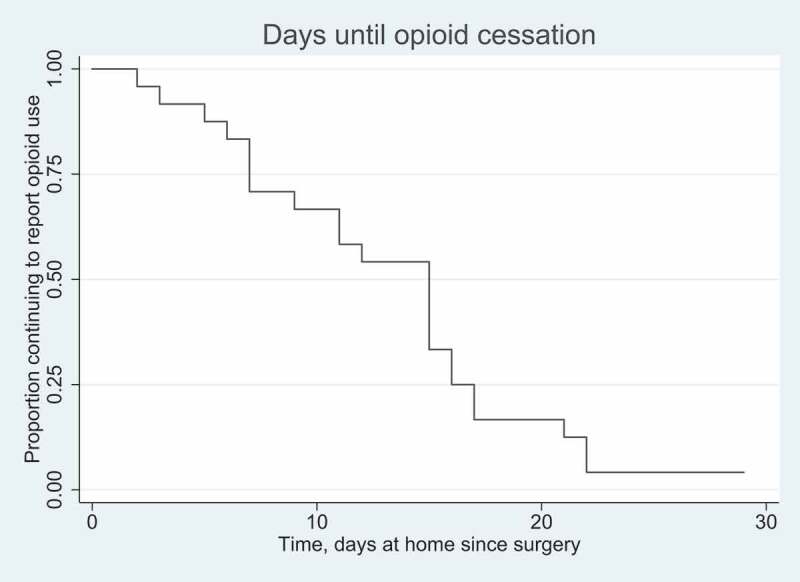
Kaplan-Meier curve of time to opioid cessation

Duration of opioid use ranged widely from 0 to 29 days, with a mean of 13 days (SD = 7.0). Only one individual reported no opioid use at home, and the last day of reported opioid use was day 29. Figure 3 shows the Kaplan-Meier curve of time to opioid cessation. The mean time to opioid cessation was 17.2 days (SD = 4.8; range = 11–22) in the CPSP group and 11.2 days (SD = 7.2; range = 0–29) in the group that did not develop CPSP (*P* = 0.16). No youth were taking opioids at 4 months.

### Subacute Pain Curve Features Associated with CPSP

The average pain severity for the first 2 weeks was 2.6 (IQR = 1.8–3.1) for those without CPSP compared to 5.3 (IQR = 5.1–5.9) for those with CPSP, and they experienced high pain severity on 2.9% (IQR = 0.0%–11.9%) of days compared to 45.9% (IQR = 27.6%–72.0%) among those who developed CPSP. Subjects without CPSP experienced fewer days with high pain interference (9.7%; IQR = 1.7%–12.9%) compared to 49.4% (IQR = 31%–60%) among those with CPSP. Youth without CPSP achieved 56% (IQR = 42%–74%) improvement in pain severity compared to 35% (IQR = 24%–41%) in youth with CPSP at the end of 30 days. Univariate analyses comparing features of the pain severity process and pain interference process by CPSP status using two-sample *t*-tests are presented in Table 2. Comparison of features from subacute pain severity and pain interference raw curve data showed that subjects without CPSP demonstrated much lower magnitude levels than their counterparts with CPSP. Pain variability and rate of change were not significantly associated with CPSP in our pilot sample.

**Table 2.** Comparisons of subacute pain curve features by 4-month pain status

Scale	Construct	Measure	Without CPSP (*N* = 19), mean (SD)	With CPSP (*N* = 5), mean (SD)	*P* values (adjusted to have 5% false discovery rate)
Pain severity (0–10)	Magnitude	14-day mean	2.6 (1.0)	5.3 (1.1)***	0.005
		30-day mean	2.0 (0.8)	4.6 (1.1)***	0.005
		% days ≥5	2.9 (6.8)	45.9 (35.7)***	0.005
	Variability	14-day SD	0.9 (0.4)	1.1 (0.3)	0.48
		30-day SD	1.0 (0.4)	1.2 (0.3)	0.33
		30-day range	3.5 (1.6)	4.3 (1.1)	0.29
	Rate of change	Linear slope	−0.08 (0.05)	−0.09 (0.03)	0.55
		30-day relative change	−0.56 (0.27)	−0.35 (0.17)*	0.08
		Difference in slope	0.07 (0.11)	0.05 (0.13)	0.72
Pain interference (0–10)	Magnitude	14-day mean	3.5 (1.4)	5.4 (1.6)*	0.06
		30-day mean	2.6 (1.2)	4.6 (1.8)*	0.07
		% days ≥5	9.7% (11.9%)	49.4 (32.5%)**	0.01
	Variability	14-day SD	1.4 (0.6)	1.3 (0.7)	0.91
		30-day SD	1.4 (0.6)	1.4 (0.4)	0.92
		30-day range	5.1 (1.9)	5.4 (1.9)	0.91
	Rate of change	Linear slope	−0.12 (0.07)	−0.11 (0.03)	0.78
		30-day relative change	−0.64 (0.23)	−0.44 (0.28)	0.18
		Difference in slope	0.19 (0.13)	0.09 (0.25)	0.34

Difference in slope = the difference between the linear slope of the first and the second 15-day periods.

*P < .05. **P < .01. ***P < .001, for unadjusted *P* value based on two-sample *t*-test.

## Discussion

In this pilot study, adolescents demonstrated adequate completion rates of daily electronic diaries over the month after discharge from surgery. We observed large differences in magnitude and rate of change of pain during subacute recovery between youth who went on to develop chronic pain compared to those who went on to recover by 4 months postsurgery. We also observed wide variability in opioid use in both groups. The more granular data allowed us to detect informative patterns in pain severity and interference processes that cannot be done with sparse data collection. Findings suggest that it may be feasible to monitor daily pain and opioid use immediately following hospital discharge from major surgery to identify youth whose pain and opioid use are not showing expected resolution.

We employed several approaches to minimize participant burden to optimize feasibility of capturing pain and function on a daily basis. This included using a single short-form measure, an online data collection system, and automated reminder e-mails. Recent data from the Pew Research Center demonstrate that 95% of adolescents have access to a smart phone and that this is consistent across sociodemographic backgrounds.^35^ This has increased from 75% in 2015, making electronic data collection via mobile devices increasingly feasible among youth. Houle et al. similarly assessed daily pain in adults recovering after musculoskeletal surgery; however, the majority of their participants completed paper diaries.^10^ Houle et al. highlighted the limitations of paper diaries, which may be completed retrospectively by participants and therefore are subject to recall bias. Other advantages of electronic data capture systems with automated reminders include convenience for patients and providers, lower potential for data entry errors, and lower administrative costs compared to paper diaries or telephone calls.^36^ Participants in our study received a small incentive for completion of their daily monitoring. It will be important to incorporate creative ways to incentivize patients to complete daily monitoring in the clinical setting; for example, access to viewing tracking data.

Among youth recovering from musculoskeletal surgery, trajectory features representing magnitude and rate of change of pain differed between those who recovered and those who developed chronic pain by 4 months. Higher pain severity as well as higher pain interference during the subacute period placed youth at significantly higher risk for chronic pain at 4 months. Interestingly, youth who went on to recover not only had greater reduction in pain across the first 30 days but also demonstrated more rapid change in the earlier portion of recovery, although this was not statistically significant in this small sample. These pilot findings build on prior pediatric studies that have demonstrated that higher pain intensity 2 weeks after surgery is associated with CPSP^3,7^ by taking a more granular approach to identify measurable features even before the 2-week mark that are associated with elevated risk for CPSP. The potential implications of these findings are twofold. First, findings suggest that we may be able to identify youth who are experiencing delayed resolution of pain early on through daily electronic monitoring of subacute pain trajectories and statistical techniques that can potentially be applied and interpreted in a clinical setting. Identification of these youth would allow direction of resources and tailoring of treatment for these youth, with the ultimate goal of preventing transition to chronic pain. Second, these findings also suggest that the initial weeks following surgery may be an important period when acute to chronic pain transition occurs, thus providing opportunity to study mechanisms during transition from acute to chronic pain. Similar short time series trajectories on activity, mood, and sleep processes may provide additional information about the recovery process and shed light on innovative interventions to reduce CPSP. Larger studies are needed to validate the predictive value of pain curve features and to examine additional trajectory features, such as nonlinear models of change in pain.

We also found wide variability in postsurgical opioid use. The opioid epidemic is a pressing public concern affecting the health of adolescents in North America. Postsurgical prescription opioid use is considered a driver of the opioid epidemic, with up to 5% of children developing persistent opioid use following surgery.^11^ Therefore, there is a great need to better balance the prescribing of opioids for management of pain at home following surgery with the risk for development of problematic opioid use behaviors. Although a mean duration of opioid use of about 2 weeks is consistent with recent literature,^29^ we found a wide interindividual difference in the number of days that patients used opioids while recovering from surgery, supporting the concept that personalized opioid prescribing may achieve a better balance between pain management and risk of overprescribing. To date, the mechanisms responsible for these large differences in opioid use remain poorly described, and there are no tools to prospectively predict which adolescents will use more opioids than their peers. Future studies should employ closer monitoring of daily opioid use in order to identify which adolescents are at increased risk for prolonged opioid use.

By design, our study focused on the recovery period immediately after hospital discharge. However, additional potential opportunities exist for identifying youth at risk for chronic pain or prolonged opioid use following surgery. These include the pre-operative period where screening of psychosocial risk factors and presurgery pain can be employed^4,28,29,37,38^ and in the hospital, where monitoring of acute pain, function, and opioid use^37,39^ may prove useful to identify those at risk for poor outcomes. Additional research is needed to determine optimal timing of screening and monitoring procedures, as well as interventions aimed at prevention during the pre- and postoperative phases of surgery.

### Limitations and Future Directions

This pilot study had little sociodemographic diversity, and participants were recruited from an ongoing longitudinal study, which may impact generalizability of feasibility findings. This study was designed to be an exploratory study to examine feasibility of daily monitoring after hospital discharge from surgery and to generate hypotheses for future research focusing on the understudied subacute recovery period. Qualitative data will be important to understand patient stakeholder perspectives of feasibility in this context. Further limitations include that patients were not physically examined to diagnose CPSP, and daily opioid use was assessed by self-report; thus, type and dose of opioid were not able to be reliably collected. The small sample size of this pilot study limited our power to conduct multivariate analyses and ability to draw definitive conclusions. Indeed, the sample was too small to examine instances of persistent opioid use and opioid misuse. Studies are also needed to understand how anesthesia and pain protocols might influence opioid and pain trajectories. However, the aim was to identify potential target features of pain trajectory data for future study. We hope that this study will encourage the field of postsurgical pain research to focus efforts on this critical period, to understand subacute pain and opioid trajectories.

## Conclusions

We found it feasible to collect daily pain data among youth recovering at home after major musculoskeletal surgery to capture features of individual subacute pain curves. Youth who developed chronic pain reported higher daily pain severity and interference and slower resolution of pain over the 30 days at home following surgery. Research is currently underway to validate subacute pain trajectories to identify risk for chronic pain after surgery in order to develop monitoring procedures and direct interventions to prevent the transition from acute to chronic pain.
